# Methods and Strategies for Biomonitoring in Occupational Exposure to Plant Protection Products Containing Glyphosate

**DOI:** 10.3390/ijerph20043314

**Published:** 2023-02-14

**Authors:** Horațiu Moldovan, Silvia Imre, Radu Corneliu Duca, Lénárd Farczádi

**Affiliations:** 1 Department of Occupational Medicine, Faculty of Medicine, George Emil Palade University of Medicine, Pharmacy, Science, and Technology, 540142 Târgu Mureş, Romania; 2 Center for Advanced Medical and Pharmaceutical Research, George Emil Palade University of Medicine, Pharmacy, Science, and Technology, 540142 Târgu Mureş, Romania; 3 Faculty of Pharmacy, George Emil Palade University of Medicine, Pharmacy, Science, and Technology, 540142 Târgu Mureş, Romania; 4 Environmental Hygiene and Biological Monitoring Unit, Department of Health Protection, National Health Laboratory (LNS), 3555 Dudelange, Luxembourg

**Keywords:** glyphosate, exposure, biomonitoring, bioanalytical methods

## Abstract

Glyphosate, and the ever growing reliance on its use in agriculture, has been a point of contention for many years. There have been debates regarding the risk and safety of using glyphosate-based herbicides as well as the effects of occupational, accidental, or systematic. Although there have been a number of studies conducted, the biomonitoring of glyphosate poses a series of challenges. Researchers attempting to determine the occupational exposure face questions regarding the most appropriate analytical techniques and sampling procedures. The present review aims to summarize and synthetize the analytical methodologies available and suitable for the purpose of glyphosate biomonitoring studies as well as discuss the advantages and disadvantages of each analytical technique, from the most modern to more well-established and older ones. The most relevant publications that have described analytical methods and published within the last 12 years were studied. Methods were compared, and the advantages and disadvantages of each methods were discussed. A total of 35 manuscripts describing analytical methods for glyphosate determination were summarized and discussed, with the most relevant one being compared. For methods that were not intended for biological samples, we discussed if they could be used for biomonitoring and approaches to adapt these methods for this purpose.

## 1. Introduction and Background—Glyphosate Use and Exposure

**Figure 1:**
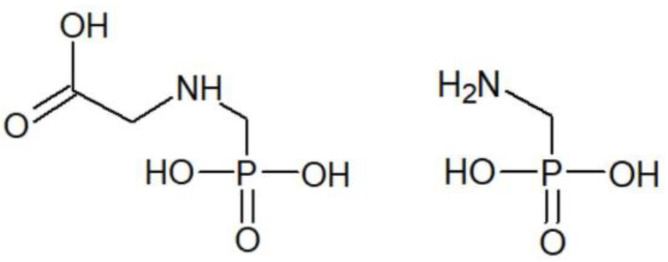
Chemical structure of glyphosate (**left**) and aminomethylphosphonic acid (**right**).

Glyphosate, also known under its IUPAC name *N*-(phosphonomethyl) glycine, while discovered by a Swiss chemist, Dr. Henri Martin [1], was initially developed as a chemical chelating agent, a chemical intermediate for the synthesis of other molecules, and as a possible bioactive compound [2]. Independently, sometime later, due to the potential of chelating metals, a number of derivatives of aminomethylphosphonic acid (Figure 1) including glyphosate (Figure 1) were studied as potential water-softening agents [1]. During the research, however, the herbicidal activity of some of these compounds was discovered, and after some study, glyphosate was discovered to be a promising candidate for such use [1]. Not long after this discovery, the first commercial formulation of glyphosate to be used as a broad-spectrum weedkiller was created [1].

Glyphosate base plant protection products are effective herbicides by inhibiting an important plant enzyme, 5-enolpyruvylshikimate-3-phosphate synthase, which is present in plants and fungi, but not in animals and humans [3]. This enzyme is part of the biological mechanism during which plants synthesize aromatic amino acids. These amino acids are essential in many ways for the survival and growth of plants; thus, glyphosate inhibits the plant from functioning normally and slowly leads to the deterioration of the plant, both overground and underground. Loss of herbicidal activity occurs through the hydrolysis of glyphosate into its main metabolite aminomethylphosphonic acid (AMPA) [3].

To improve the efficacy of glyphosate, modified crops have been developed, which are resistant to glyphosate by genetically engineering plants to express genes from a type of bacteria, after discovering that it contained a form of the enzyme 5-enolpyruvylshikimate-3-phosphate synthase, which is not inhibited by glyphosate [4]. This made the use of glyphosate the first choice for crop farming as it facilitated the destruction of unwanted weeds without affecting crops.

Glyphosate is a polar, readily water-soluble compound; it tends to partition in water versus air and easily absorbs into the soil particles. During application, small quantities of aerial drifts, splash, or drip can cause harm to non-target surrounding plants. Glyphosate is highly chemically stable in water in a wide pH-range and is not photosensitive to degradation from sunlight. The main path of the decomposition of glyphosate in water and soil particles is through microbial degradation and is dependent on the type and number of microorganisms [3].

Due to its wide use in recent years, concerns have grown with regard to its toxicity and the health risks involved with exposure to glyphosate and glyphosate-based herbicides [5]. While some regulatory agencies consider that it does not pose a risk to public health and that it is unlikely to be carcinogenic to humans [6,7], others have concluded that it is a “probably carcinogenic” substance to humans [8]. Most regulatory authorities, however, have concluded that it is necessary to limit the human intake of glyphosate. Although it is a controversial topic and it is still debated whether it has a role as a tumor cell initiator or promoter, and studies are still ongoing, there are results that have shown the cytotoxicity, involvement in genetic damage, and even some tumor promoting activity of glyphosate [9,10].

In 2017, the license for glyphosate use in the European Union was renewed for five more years until December 2022, after the previous 15-year license had expired, causing a controversial and highly divisive debate [11]. As glyphosate residues have been detected in food, groundwater, and even drinking water [12,13], most regulatory agencies around the world, even those that have classified it as posing no risk to public health, have imposed limits on the exposure and intake for humans, currently at 1.75 mg/kg bw/day in the USA and 0.5 mg/kg bw/day in the EU (increased in 2015 from 0.3 mg/kg bw/day) [13,14,15]. Some independent scientists, however, consider these limits to be too high, suggesting an acceptable daily intake of 0.1 mg/kg bw/day or less [16].

Biomonitoring in glyphosate exposure is not only a challenge, but is should also be a must for both the occupational and non-occupational exposed population, considering the high amount of usage worldwide. For example, in the European Union, the total glyphosate sales in 2017 reached 44,250 tons, accounting for a proportion of 34% of all herbicides [17]. It is estimated that around 11–13% of agricultural workers are exposed to glyphosate [18], which makes the need for glyphosate biomonitoring of even greater need.

In a series of 13 acute glyphosate poisonings, Zouaoui et al. [19] described the associated symptoms such as respiratory alteration, oral and pharynges ulceration, hepatic and renal toxicity, cardiac arrest, laboratory parameters disturbed, and many other affected organs. The authors indicated a mean value of 61 mg/L glyphosate in blood in mild–moderate intoxication, a mean concentration about fourteen times greater in severe intoxication, and sixty-eight times higher in mortal cases.

During a study performed on farmers of different crops in Thailand, Wongta et al. found that the occupational exposure of the farm worker groups led to significant, quantifiable levels of glyphosate in the urine of a large percentage of them compared to a control group that showed no detectable urinary glyphosate [20]. Urine concentrations were between an average of 2.01 ng/mL and 3.11 ng/mL for the different types of farmer groups and was thought to be worsened by the lack of protective equipment for the farm workers. Similar previous studies performed by researchers in this group have also shown that the occupational exposure of farm workers from other regions of Thailand have similar quantifiable glyphosate levels in urine [20].

A study by Melissa Perry et al. on historical urine samples collected decades prior from American farmers exposed to glyphosate also showed that compared to non-users who had no detectable traces of glyphosate in their urine, the farmers actively using glyphosate on their crops, for the most part, had quantifiable levels of urinary glyphosate with an average of 4.04 μg/kg urine, and as high as 12.0 μg/kg urine [21].

The need to determine the exposure of humans to glyphosate and glyphosate based-herbicides has thus become more important than ever before, in order to correctly assess the risk it poses for humans.

## 2. Difficulties in Laboratory Assessment of Glyphosate—Ongoing Research

**Figure 2:**
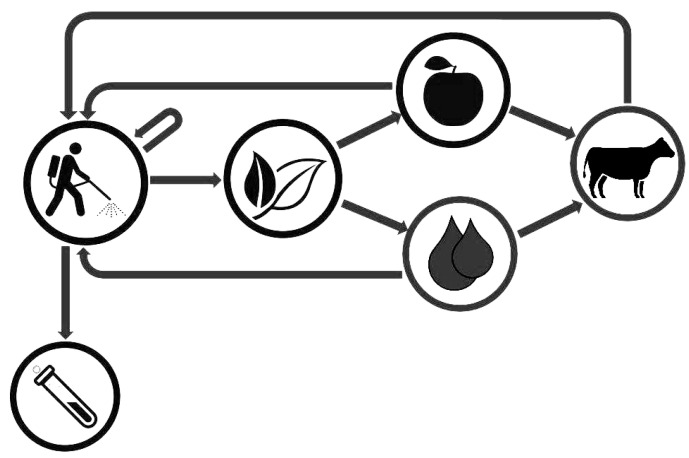
Sources of exposure to glyphosate [23,24].

Rapid and reliable analytical techniques are essential in clinical chemistry because they enable timely and precise diagnosis and appropriate treatment [22], and this is essential in the field of occupational medicine for a proper biomonitoring strategy in occupational exposure to glyphosate. Occupational exposure to glyphosate can have the direct pathway through inhalation or topic absorption during the application of herbicide products based on glyphosate, but there can also be a secondary source of exposure through drinking water and food contaminated with glyphosate (Figure 2).

There are many types of analytical techniques that can be used for the detection and quantification of glyphosate and similar compounds. For environmental and food analysis (water, soil, plants), the most widely used techniques involve liquid chromatography, for example, high pressure liquid chromatography (HPLC) with either ultraviolet detection (UV) or fluorescence detectors (FLD) and liquid chromatography coupled with mass spectrometric detection (LC-MS or LC-ICP-MS), but methods also exist that use ion chromatography (IC) [22,25,26,27,28,29,30,31,32,33,34,35,36,37,38,39,40,41,42,43]. For bioanalytical and biomonitoring purposes, methods for glyphosate determination in biological matrices (urine, blood, plasma, serum) described in the literature have mostly used liquid chromatography techniques coupled with different types of detection (ultraviolet, fluorescence, but most often mass spectrometry) [19,21,36,37,38,39,44,45]. Other techniques such as gas chromatography (GC), which is also frequently coupled with mass spectrometry (GC-MS), ion chromatography coupled with mass spectrometric detection [46], and the enzyme-linked immunosorbent assay (ELISA) have also been used for glyphosate determination [21,25]. More recent developments include the tentative use of electrochemical sensors for the detection of glyphosate from different types of matrices [36].

Choosing the right technique and developing a suitable method, however, might not always be straight-forward, and it is important to choose the correct technique depending on the exact application, as each technique has its own advantages and drawbacks. Likewise, regardless of the technique, the method must be optimized for the application to ensure the proper sensitivity, selectivity, and robustness of the measurements and thus obtain accurate and reliable results. One must also always take into account the availability of the equipment and performance of the equipment that is available as well as the cost of the analytical determination and funding available. Some techniques might offer better sensitivity, selectivity, and/or robustness, which, depending on the application, might not even be necessary, but require better, more expensive equipment, longer sample preparation times, and can lead to increased costs.

## 3. Scope, Methods, and Results

An integrated and synthesized overview of the current state of knowledge in the methods and strategies for biomonitoring in occupational exposure to glyphosate is proposed.

The different types of approaches used, their results, the advantages, and disadvantages of the most often used techniques are discussed to pinpoint some of the most important considerations when developing a method for glyphosate and AMPA identification and quantification, depending on the application in question, in order to provide an efficient and useful tool for researchers studying occupational exposure to glyphosate, which in increasingly of interest.

For the current review, the most relevant publications in the scientific literature, published within the last 12 years, were selected by using internationally recognized databases such as the PubMed and Web of Science platforms. The search was conducted for terms such as ‘Glyphosate’, ‘AMPA’, ‘Glyphosate biomonitoring’, and ‘AMPA biomonitoring’. These search terms were also conducted with the inclusion of ‘AMPA’. The most recent search was performed on 30 September 2022. Compared to other reviews regarding glyphosate biomonitoring, manuscripts also describing environmental matrices (food residues, water or soil) were included. Publications were not reviewed for their quality or ranked on the basis of other studies [47].

Only research papers that described the analytical methods for identifying glyphosate in biological matrices were analyzed as well as papers describing different methodologies for sampling in exposure to glyphosate (Table 1). The current review mainly focused on the challenges in choosing and developing appropriate analytical methods in occupational exposure biomonitoring.

Combining these two approaches, the aim was to identify inconsistencies in the prior results and the potential explanations, strengths, and weaknesses of different approaches to describe existing gaps and future research directions for a suitable strategy to biomonitor the occupational exposure to glyphosate.

To achieve this, descriptive analysis and data extraction into standardized templates for the most relevant strategies for sampling and biomonitoring exposure were used.

## 4. Discussion

As previously stated, there are many types of methods for the determination of glyphosate described in the literature. Regardless of the technique used, as a general rule, it is important that the method used is validated with regard to the important and relevant performance parameters, both in the case of methods developed in-house or purchased as a ready to apply kit.

It is quite obvious from the articles studied, and also when looking at a general cross section of the scientific literature regarding glyphosate quantification and biomonitoring, that although various approaches and methods have been applied, there are three main points of focus when discussing glyphosate determination: the matrix from which to perform the determinations, the sample preparation method, and the analytical technique to be used.

### 4.1. Sample Matrix Selection

The foremost consideration when selecting which matrix to determine glyphosate and/or its metabolite (AMPA) from is of course the importance and relevance of the samples that will be collected for the purpose of each study. For biomonitoring purposes, while blood or serum have been used, urine is considered more suitable as glyphosate is mainly excreted unchanged through urine, where it yields higher concentrations [14]. Furthermore, urine is more easily obtainable as its collection is non-invasive when compared to blood or breast milk collection, and thus can increase the willingness of the subjects to participate in biomonitoring studies.

Another advantage of urine samples in comparison to blood samples is that generally, when using urine, a less complex matrix compared to blood, there is a reduced matrix effect in the case of some analytical techniques that are susceptible to this effect as well as a reduced ion suppression when using LC-MS methods. This can not only simplify and reduce the cost of the sample preparation and cleanup techniques that need to be applied, but can also lead to less sensitive or selective analytical techniques being applicable for analysis, or measurements carried out on older or less powerful equipment. However, due to the chemical properties of glyphosate and AMPA, sample cleanup and preparation still usually imposes complex extraction procedures and derivatization reactions.

Aside from the selection of the biological matrix for biomonitoring, it is also helpful and relevant to further determine the source and the level of exposure, especially in cases where there is no obvious exposure to the substance. When possible, an approach where environmental and/or food matrices are also measured to determine exposure is the best course of action. This has been the approach in a number of studies reported in the literature, where in general, drinking water has also been sampled and measured to determine the glyphosate content, but groundwater and food samples (vegetables for example) could also yield important data. This is also true when discussing occupational exposure as aside from the known exposure, there can be other, secondary sources of exposure [35,55]. Environmental matrices have the advantage of generally being measurable by the same technique and method as urine or blood samples, requiring only some adaptations to the sample preparation method. As water (ground or drinking) is a chemically less complex matrix when compared to biological matrices, and is less likely to be susceptible to the matrix effect or analyte recovery problems, the analysis of glyphosate from water usually requires a less tedious and complex sample preparation method [32,33] compared to urine or blood [44,48], but there are methods using LC-MS for which sample clean-up is also quick and protein precipitation is simple [40,50,52]. This is not to say that glyphosate determination from drinking or groundwater is simple or straightforward, but if the study includes both environmental and biological matrices, it is generally a good approach to start in-house method development for the chemically simplest matrix (e.g., drinking water) and further develop and adapt the methodology for more complex matrices. There are many instances where methods developed and previously used for environmental or food matrices have been adapted for use as biological samples or vice-versa such as in the case of Krüger et al. or Connolly et al. [46,48]. There have been cases when ELISA methods have been described in the literature as being more susceptible to the matrix effect when analyzing drinking water compared to urine (due to the higher metal ion content or chlorination of the drinking water) [14], however, ELISA, being a technique that routinely uses kits that have been previously verified by the manufacturers, these issues, if present, should be known beforehand.

Thus, in order to correctly assess the pathways through which glyphosate can cause harm to human health, analytical methodologies for the determination of glyphosate need to be put in place for detection and quantification from environmental samples (soil, ground water), food samples (drinking water, crops, vegetables etc.) in addition to biological samples from test animals and humans in different biological matrices (blood/plasma, urine, tissues etc.). For glyphosate exposure specifically, as glyphosate and its metabolite are eliminated through urine, this matrix is a better choice than blood, and is as relevant for biomonitoring, if not more relevant, as blood or plasma, but has the advantage of easier, non-invasive collection, simpler processing and storage conditions, and the high stability of glyphosate and AMPA over prolonged periods of time. It can simplify the participation of subjects in a study, increase or make compliance with international regulations easier, but also has the added advantage of cost reduction in both sample collection as well as due to the higher concentrations of glyphosate and AMPA to be expected in urine compared to blood, which can result in simpler and more cost-effective sample preparation and/or the use of somewhat less powerful analytical equipment. Selecting urine as the matrix is thus an important aspect as the analytical methods and measurement already necessitate expensive reagents, consumables, techniques, and equipment. Of course, if accumulation studies or more detailed pharmacokinetic studies are needed to be carried out, it might not be sufficient to only measure the urine concentrations of glyphosate and AMPA, but for biomonitoring studies, it generally suffices.

### 4.2. Sample Preparation Method

When choosing the sample preparation and cleanup methodology to be used, there are two main considerations that need to be taken into account. First is the sample type itself, as sample preparation needs to be adapted to the chemical complexity of the matrix to be analyzed and other compounds expected to be present in the samples, aside from the analyte of interest.

The second important consideration is the analytical technique to be used to perform the determinations as some techniques might be more susceptible to interference from other chemical compounds in the samples, leading to the matrix effect, loss of sensitivity, and other issues. Furthermore, depending on the technique used, the detection of glyphosate might not even be possible without performing a chemical derivatization due to the chemical properties of the molecule (Table 1). Techniques that inherently need some type of derivatization of glyphosate in order to be used are fluorescence detection in HPLC or simple spectrofluorimetry, gas chromatography (regardless of whether flame ionization detection (FID) or MS detection is used), and in certain cases, ELISA [12,48]. This can introduce extra costs, involve lengthier processes, and can sometimes lead to a drop in the robustness and or/sensitivity of the methods. In some cases, the sample preparation and cleanup method must account for the sensitivity of certain types of techniques and equipment for some compounds, which then need to be removed from the samples in order to avoid deterioration of the equipment or certain parts and consumables relating to these equipment (e.g., removing macromolecules and solid particle when using chromatographic techniques, removing metal ions when using mass spectrometry).

Generally, the selection of the sample preparation and cleanup method goes hand in hand with the selection of the analytical methodology, as each one is highly dependent on the other, and the choice of both is influenced by the equipment available. Therefore, the sample preparation steps should not only be amended to the analytical equipment available to be used, but also to the auxiliary equipment at the disposal of the researchers (e.g., centrifuges, liquid–liquid extractors, solid phase extractors, incubators, multichannel pipettes, etc.).

Another important aspect of sample preparation and cleanup is the cost and time needed to perform the procedure. If possible, it is preferred that the sample preparation process is simple and rapid in order to facilitate the analysis of as many samples as possible in a short period of time. Lengthy and complex sample preparation processes are best avoided if not absolutely necessary or if they can be replaced with simpler methods to achieve sample cleanup.

Although a simple sample preparation method is preferred and can have the added benefit of cost reduction, it is not always applicable and, as previously stated, it is highly dependent on the type of sample to be analyzed and the analytical techniques available. Conversely, when possible, and the researchers have a choice, the aspect of sample preparation can influence the selection of the analytical technique to be used for analysis and even the selection of the biological matrix for the biomonitoring process.

For the glyphosate and AMPA analysis, extraction from biological fluids and samples can be a tough challenge due to the highly hydrophilic nature of these small molecules. If the analytical method necessitates the derivatization of glyphosate and AMPA for the detection of the compounds, as is the case when using gas chromatography or liquid chromatography with UV or fluorescence detection, the derivatization process (e.g., 2,2,2-trifluoroethanol, fluorenylmethyloxycarbonyl chloride, etc.) usually results in derivates of glyphosate and AMPA, which are more hydrophobic in nature and thus allows for easier extraction from aqueous matrices such as urine or plasma, using either liquid–liquid extraction using an organic solvent (e.g., ethyl acetate or tetra-hydrofuran), or by solid-phase extraction with appropriate silica based (e.g., C18) extraction cartridges designed for hydrophobic compounds. If the analytical technique used for the quantification of glyphosate and AMPA does not necessitate derivatization for the detection of the compounds, as is the case with LC-MS, derivatization can be still performed in order to facilitate the extraction of the analytes from biological matrices. The derivatization of glyphosate and AMPA will also facilitate analytical LC separation using C18 type reverse phase chromatography columns, which are more robust and simpler to use and maintain compared to hydrophilic liquid chromatography columns. At the same time, in certain situations, the derivatization of glyphosate and AMPA can improve the selectivity of the mass spectrometric detection by allowing for more specific fragmentation patterns. For LC-MS analysis, glyphosate and AMPA are sometimes also derivatized using fluorenylmethyloxycarbonyl chloride (FMOC-Cl) [30], dansyl chloride [56], or trimethyl orthoacetate [54] in order to make the analyte extraction and analytical separation easier, however, there are also a large number of methods using LC-MS to measure the underivatized glyphosate [32,36,50,51,52]. While it has many advantages when trying to measure glyphosate and AMPA in biomonitoring studies, derivatization involves additional costs and time to be performed and the yields of the derivatization reactions might induce a variation, while the extraction can reduce the sensitivity as well as the reproducibility of the entire analysis method. For underivatized samples measured through mass spectrometry (LC-MS or IC-MS), clean-up needs to be performed using a simple protein precipitation step [36,50,52] or hydrophilic–lipophilic balanced solid phase cartridges designed for hydrophilic compounds such as glyphosate and AMPA. The analytical columns also need to be selected accordingly in order obtain the retention of underivatized glyphosate and AMPA, which need hydrophilic interaction (HILIC) analytical columns (which are generally more expensive and less robust than C18 columns), and there do exist special LC columns designed for underivatized small polar molecules (e.g., glyphosate and AMPA) such as the Acclaim Trinity Q1 or Torus DEA columns. In the end, the choice of sample preparation method depends on the analytical method and experience of the analysts, as there are detection methods that only work through the derivatization of glyphosate and AMPA, but if the method used allows for the detection of these analytes underivatized, the choice depends on the analyst developing the method: some analysts might choose to derivatize the compounds to make sample extraction and analytical separation easier (recommended for less experienced analysts), while other analysts might prefer to reduce the costs and time of sample preparation by avoiding derivatization.

### 4.3. Analytical Technique

As previously discussed, deciding on a suitable analytical technique is highly dependent on the type of sample to be analyzed and the equipment available for both the analytical measurements themselves and for the sample preparation. Some analytical techniques need certain specific sample preparation steps, but it is necessary to take into consideration a point of view including the technique, cost, and time cost.

The enzyme-linked immunosorbent assay (ELISA) is a technique used mostly, but not exclusively, for biological samples. It has the advantage of being a simple method including simple sample cleanup and preparation, with generally good sensitivity and selectivity, but is mostly performed using kits manufactured specifically for certain types of determinations. This makes it not only a method that is highly reliant on the availability and price of the kits, but also one that offers less flexibility. ELISA kits are usually verified for performance by the manufacturer for certain applications, but accounting for every variable that can occur in samples, especially complex biological matrices, is near impossible to achieve and in some cases, it is recommended that the end users verify and validate certain aspects themselves. Thus, unexpected and unforeseen interferences and lack of performance might appear in some cases (e.g., matrix effect in drinking water) when using ELISA kits [14], and even some manufacturers recommend the use of alternative methods for the confirmation of results in certain situations [57].

Gas chromatography (GC) is another popular technique used in glyphosate detection. Due to the lack of the volatility of glyphosate and its metabolites, however, sample preparation always imposes a derivatization process, with derivatization agents such as 2,3,3,4,4,4-heptafluoro-1-butanol, 2,2,2-trifluoroethanol, *N*-methyl-*N*-(tert-butyldimethylsilyl) trifluoroacetamide with 1% tert-butyldimethylchlorosilane, or other reagents [22,48,53]. This is an important consideration both due to the added cost, time, and equipment needed to perform this step. Gas chromatography is frequently used in tandem with mass spectrometric detection in order to provide suitable selectivity and sensitivity, and at the same time, generally offers good accuracy and robustness for the measurements being performed.

Liquid chromatography (LC) is, however, the most often used analytical technique for glyphosate determination. It offers excellent flexibility and is widely available in many laboratories around the world, being used for many purposes over a wide range of applications (clinical, forensic, pharmaceutical, environmental, food, environmental, etc.). Liquid chromatography can be coupled with many types of detectors (ultraviolet-visible (UV–Vis), fluorescence (FLD), refractive index (RID), mass spectrometry, etc.) and a number of these can be used for glyphosate detection and measurement. UV and fluorescence detection, although previously used and reported in the literature [25,58,59,60], has largely been replaced with mass spectrometric detection, as mass spectrometry offers superior sensitivity and selectivity. Furthermore, while fluorescence and UV necessitate specific types of sample derivatization in order for glyphosate to be detectable with these types of detectors, with the widely used derivatization reagents being fluorenylmethyloxycarbonyl chloride [30] for fluorometric detection, and 4-chloro-3,5-dinitrobenzotrifluoride or p-toluenesulfonyl chloride for ultraviolet detection [34,59]. Mass spectrometry has the added advantage that detection can be performed for both underivatized glyphosate as well as glyphosate chemically transformed with different derivatization agents such as fluorenylmethyloxycarbonyl chloride, trimethyl orthoacetate, or dansyl chloride [30,54,56]. This offers LC-MS methodologies the added benefit of being highly flexible, allowing for many types of sample preparation methods, while offering the advantage of various types of analytical separation mechanisms that are available for liquid chromatography applications. Therefore, sample preparation can be developed and adapted more easily with the available auxiliary equipment, cost, time restraints, and sample types which must be analyzed bearing in mind that this means that there may be many sample types that can be analyzed. Mass spectrometry, due to its molecular mass-based selectivity, can also differentiate between different chemical compounds when not (completely) chromatographically separated, while UV and FLD detection cannot. This offers a possibility for shorter analysis run-times for each sample as the analytical separation of different matrix compounds might not always be completely achieved, but one must verify that the lack of separation does not cause ion suppression or matrix effects. Depending on the type of LC-MS equipment (triple quadrupole, ion trap, quadrupole time-of-flight, orbitrap, etc.), the different types of ionization mechanism can also help improve the selectivity and sensitivity of the method. The drawback of LC-MS, however, is the high cost of the equipment as well as the high costs of continuous maintenance when compared to other techniques. Due to this, the availability of the technique and/or trained personnel can also pose a problem if multicentric studies are being performed as well as the fact that in the case of such multicentric studies, because of the different performances between the different types of mass spectrometers, inconsistencies in the sensitivity, selectivity, and robustness of the measurements can occur between laboratories, which can cast doubt on the obtained results.

Other methods that have been described for the measurement of glyphosate use ion chromatography (IC), sometimes coupled with mass spectrometry [36,37], and inductively coupled plasma mass spectrometry (ICP-MS) [35], but these techniques are not as widely available and versatile as LC-MS or GC-MS. Although they have limited usability and availability, these techniques can be used when already available, as they only require minimal sample preparation and clean-up, thus being simple and cost-effective.

While LC-MS is the generally preferred analytical technique for the biomonitoring of glyphosate exposure as it allows for flexibility in sample preparation as well as high sensitivity and selectivity, it might not be a technique readily available in all laboratories due to the acquisition and maintenance costs, which are much higher for LC-MS equipment compared to liquid chromatographs coupled with UV or fluorescence detectors, and even GC-MS systems. If available, however, it is the go-to technique for biomonitoring glyphosate exposure.

## 5. Conclusions

When it comes to biomonitoring studies in occupational exposure to glyphosate, there are many approaches, each having their own advantages and disadvantages. The most important aspect when performing biomonitoring is to select the biological matrix that is most appropriate and relevant to glyphosate exposure, but to also consider the simplicity of sample collecting. Urine samples seem to fulfill both criteria and are thus a good choice to biomonitor the exposure to glyphosate.

At the same time, the analysis of environmental samples such as water can shed light and offer additional information on other (secondary) sources of exposure and should also be taken into account if deemed relevant for the biomonitoring of the workers’ exposure as they can usually be analyzed using the same methodology as that used for urine samples with small or no modifications needed.

The choice of analytical methodology for glyphosate and AMPA identification in biological matrices should be made bearing in mind the type of equipment available for both analytical measurements and the sample preparation procedures, the number and types of samples that need be analyzed, the possible time and cost constraints as well the performance necessary to obtain relevant and reliable results. LC-MS is a good technique for this purpose as it offers high sensitivity, selectivity as well as flexibility, but it might not be as widely available or is too costly to be used. Liquid chromatography coupled with UV or fluorescence detection can also be used if mass spectrometry is unavailable and might yield satisfactory results, but one must consider if the reduced sensitivity and selectivity are still sufficient for performing the measurements needed for a given study. Gas chromatography coupled with mass spectrometry is also a good option to perform biomonitoring in glyphosate exposure studies, but, just as in the case of UV or fluorescence detection, requires more steps during sample preparation. For the ELISA analysis of glyphosate and AMPA, which is highly dependent on the availability of kits, although sample cleanup and preparation is typically simple, it is recommended that the results are confirmed using other techniques such as LC-MS or GC-MS. IC, IC-MS, and ICP-MS are other techniques that can be used when readily available, but are not as widespread as other techniques such as LC-MS or GC-MS.

LC-MS is not only the most powerful technique with regard to sensitivity and selectivity for glyphosate determination, but it also allows for flexibility with regard to sample preparation. The choice of measuring underivatized or derivatized glyphosate and AMPA using LC-MS depends mostly on the experience and/or preference of the analyst. If using techniques other than LC-MS, sample derivatization and thus also extraction (solid phase or liquid-liquid) are necessary. Previous experience shows that derivatization reactions can be cumbersome and result in low yields, while LC-MS allows for the quantification of glyphosate and AMPA, even after sample clean-up as simple as protein precipitation, and without derivatization.

Regardless of the technique chosen, in order to assure reliable results, it is important that any methodology for glyphosate biomonitoring is validated before using it for measurements of the study samples. The robustness, selectivity, and simplicity of a method are all important in conferring a high level of confidence to the results obtained. At the same time, it is of advantage to perform sample preparation that is as simple as possible, and has a short analysis run-time for each sample, thus reducing the overall cost. A robust, simple, standardized, high-throughput method has the additional advantage of making it easier to transfer the method between different laboratories in multicenter studies, making it easier to implement larger, more expansive biomonitoring studies.

## Figures and Tables

**Table 1:** Methods described in the scientific literature for biomonitoring glyphosate exposure from human biological samples.

Biological Matrix	Sample Timing	Analytical Separation and Detection Method	Sample Preparation Technique	Biomarker of Exposure	Limit of Quantification	Limit of Detection	Authors, Year, Journal
Urine	Once	ELISA	Dilution with water and centrifugation	Glyphosate	0.13 ng/mL	0.05 ng/mL	[12] Rendón-von Osten, Dzul-Caamal; 2017; *International Journal of Environmental Research and Public Health*
Serum, urine	Once	GC-MS	(1) Spin column extraction (2) Derivatization with MTBSTFA + 1% TBDMCS	Glyphosate	0.5 μg/mL	0.1 μg/mL	[22] Saito, Aoki, Namera, Oikawa, Miyazaki, Nakamoto, Inokuchi; 2011; *Analytical Sciences*
Serum	Once	IC-MS	(3) Protein precipitation	Glyphosate and AMPA	2 ng/mL for glyphosate 4 ng/mL for AMPA	0.6 ng/mL for glyphosate 1.2 ng/mL for AMPA	[36] Zhang, Liu, Huo, Sun, Zhang, Zhu; 2021; *Microchemical Journal*
Serum	Once	LC-MS	(4) Protein precipitation and LLE extraction of interferents with hexane	Glyphosate	25 ng/mL	not specified	[40] López-Ruiz, Romero-González, Garrido Frenich; 2020; *Journal of Pharmaceutical and Biomedical Analysis*
Urine	Once	LC-MS	Solid phase extraction (SPE)	Glyphosate	Single 20 ng/mL standard	0.5 ng/mL	[44] Connolly, Jones, Galea, Basinas, Kenny, McGowan, Coggins; 2017; *International Journal of Hygiene and Environmental Health*
Urine	Once	GC-MS and ELISA	(1) Molecular weight cutoff ultrafiltration for both GC-MS and ELISA (2) Additionally, derivatization with 2,2,2-trifluoroethanol for GC-MS	Glyphosate	Not specified for ELISA 0.3 ng/mL for GC-MS	0.05 ng/mL for ELISA 0.1 ng/mL for GC-MS	[48] Krüger, Schledorn, Schrödl, Hoppe, Lutz, Shehata; 2014; *Journal of Environmental & Analytical Toxicology*
Urine	Multiple	LC-MS	Not detailed	Glyphosate	2 ng/mL	1 ng/mL	[49] Mesnage, Moesch, Le Grand, Lauthier, Spiroux de Vendômois, Gress, Séralini; 2012; *Journal of Environmental Protection*
Blood, urine, gastric content	Once	LC–MS	Protein precipitation and sample backwashing	Glyphosate	1 μg/ml	0.1 μg/ml	[50] Tsao, Lai, HC Liu, R Liu, Lin; 2016; *Journal of Analytical Toxicology*
Urine	Once	LC-MS	Not detailed	Glyphosate	0.08 ng/mL for water 0.5 ng/mL for urine	0.02 ng/mL for water 0.1 ng/mL for urine	[51] Parvez, Gerona, Proctor, Friesen, Ashby, Reiter, Lui, Winchester; 2018; *Environmental Health*
Breast milk, urine	Once	LC-MS	Protein precipitation	Glyphosate and AMPA	0.1 ng/mL for glyphosate 0.1 ng/mL for AMPA	0.02 ng/mL for glyphosate 0.03 ng/mL for AMPA	[52] McGuire, McGuire, Price, Shafii, Carrothers, Lackey, Goldstein, Jensen, Vicini; 2016; *American Journal of Clinical Nutrition*
Breast milk	Once	LC-MS and GC-MS	(1a) Molecular weight cutoff ultrafiltration for LC-MS (1b) cation exchange cleanup prep column (2) derivatization with 2,2,3,3,4,4,4-heptafluoro-1-butanol for GC-MS	Glyphosate	1 ng/mL for both LC-MS and GC-MS	not specified	[53] Steinborn, Alder, Michalski, Zomer, Bendig, Aleson Martinez, Mol, Class, Costa Pinheiro; 2016; *Journal of Agricultural and Food Chemistry*
Plasma	Once	LC-MS	Derivatization with trimethyl orthoacetate	Glyphosate and AMPA	50 ng/mL for glyphosate 50 ng/mL for AMPA	20 ng/mL for glyphosate 20 ng/mL for AMPA	[54] Ohara, Yoshimoto, Natori, Ishii; 2021; *Nagoya Journal of Medical Science*

## Data Availability

Not applicable.
